# Enhancing Molecular Diagnostic Accuracy in Genetic Eye Disorders Through a Personalized Re-Evaluation Strategy

**DOI:** 10.1167/iovs.67.1.62

**Published:** 2026-01-30

**Authors:** Pedro Moreira Martins, Inês Figueiredo, Nuno Cruz, Beatriz Rodrigues Gaspar, Rufino Silva, Ana Luísa Carvalho, João Pedro Marques

**Affiliations:** 1 Department of Ophthalmology, Unidade Local de Saúde de Gaia e Espinho, Gaia, Porto, Portugal; 2 Department of Ophthalmology, Hospitais da Universidade de Coimbra (HUC), Unidade Local de Saúde de Coimbra (ULS-C), Coimbra, Portugal; 3 Clinical Academic Center of Coimbra (CACC), Coimbra, Portugal; 4 Medical Genetics Department, Hospital Pediátrico de Coimbra (HPC), Unidade Local de Saúde de Coimbra (ULS-C), Coimbra, Portugal

**Keywords:** inherited retinal dystrophies (IRDs), variant reclassification, whole exome sequencing, next generation sequencing (NGS)

## Abstract

**Purpose:**

Although genetic testing is crucial to provide prognostic information in genetic eye disorders (GEDs), up to 35% of cases remain unsolved after testing. We aim to describe how a tailored re-evaluation strategy, using up-to-date molecular testing, case-by-case review, and integration of in silico tools may increase the diagnostic yield.

**Methods:**

A total of 988 individuals from 800 families evaluated at the largest Portuguese GED referral center (Hospitais da Universidade de Coimbra) were included. All had a clinical diagnosis based on clinical findings and deep phenotyping and underwent genetic testing. Targeted re-evaluation included updated Next Generation Sequencing (NGS) panels, Exome Sequencing (ES) reanalysis, and assays for intronic variants, copy number variants, and ORF15-RPGR region. Variants of uncertain significance (VUS) were reassessed using segregation studies, literature/database review, and bioinformatic tools.

**Results:**

Initial testing yielded a molecular diagnosis in 475 families (59.4%). Variant reclassification enabled 39 additional diagnoses. A personalized re-testing strategy resolved 43 more, either through updated NGS panels (11), ES-reanalysis (14) and identification of deletions, GC-rich, and intronic variants (22). Overall, the solved rate increased from 59.4% to 70.1%. An additional 45 families (5.6%) carry phenotype-consistent VUS that could not be reclassified at the present date (partially-solved cases).

**Conclusions:**

This personalized re-evaluation strategy, incorporating state-of-the-art genetic testing, evidence-based variant reclassification, and case review by a multidisciplinary team increased the solved rate by over 10%. Consequently, 86 additional families received a definitive diagnosis and accurate genetic counseling, underscoring the impact of periodic re-evaluation and continuous improvement in genetic testing methodologies.

Genetic eye disorders (GEDs), such as inherited retinal dystrophies (IRDs) and hereditary optic neuropathies (HONs), are a heterogeneous group of rare, genetically inherited disorders that lead to progressive retinal and optic nerve disfunction, atrophy, and vision loss.^1^^,^^2^ Although its exact global incidence is unknown, it is estimated that approximately 1 in 3000 individuals (totaling 5–6 million cases worldwide) present some type of IRD, of which retinitis pigmentosa (RP) is the most common subtype.^2^^–^^5^ Given its early onset, they are considered one of the main causes of visual impairment in working age adults; in fact, in the England-Wales region, IRDs have surpassed diabetic retinopathy as the main cause of vision loss in this demographic, posing a significant socioeconomic health burden.^6^^,^^7^

The genetic landscape of IRDs is vast. Moreover, it is expected that more than one third of individuals worldwide are healthy carriers of IRD-associated variants in genes causing autosomal recessive disease.^3^ There has been an exponential growth and understanding of core genetics of IRDs and, to this date, 335 genes (RetNet, https://web.sph.uth.edu/RetNet/; July 29, 2025) have been identified. Yet, the diagnostic accuracy of genetic testing is still not optimal, and despite continuous technological advances, many cases remain genetically unsolved. Next Generation Sequencing (NGS), which has clearly surpassed traditional testing such as karyotyping, array comparative genomic hybridization (aCGH) studies, or Sanger sequencing, has been reported to provide a diagnostic yield of 53% to 61% in different reports and systematic reviews, meaning that almost half of the patients are left without a definite diagnosis.^8^^,^^9^

The genetically solved rate can increase by 10% to 20% with Exome Sequencing (ES), which is increasingly used as baseline testing due to its advantageous cost-benefit.^8^^,^^10^^–^^12^ Still, it poses limitations given its less than 100% coverage of the exome (especially in repetitive or GC-rich regions), modest efficacy in detecting complex structural alterations, and lack of sequencing of non-coding intron regions.^13^^–^^19^

With the increasing depth of human genome sequencing, the number of detected variants has risen substantially, and approximately one in five IRD cases present with at least one variant of uncertain significance (VUS) during genetic testing. Interpreting these variants remains challenging; re-evaluation of phenotype according to genotype, familial segregation analysis (FSA), functional studies, and review of newly published evidence can support reclassification.^20^ Cross-referencing of variant data in databases, such as gnomAD and ClinVar, may also enhance diagnosis.^21^^,^^22^ Additionally, several bioinformatic tools for functional impact prediction have been incorporated in VUS interpretation workflow, providing valuable information regarding their impact on protein function.^23^

In this study, we analyze the baseline diagnostic yield of genetic testing in the largest IRD referral center in Portugal, and aim to describe how a targeted re-evaluation strategy, incorporating detailed clinical phenotyping, the most up-to-date molecular testing, familial segregation analysis, review of published literature, and integration of in silico tools, can refine variant interpretation, increase the rate of definitive diagnoses, and ultimately improve the quality of genetic counseling offered to patients and relatives.

## Methods

### Study Design and Cohort Description

This cross-sectional study was conducted at the GED clinic of Hospitais da Universidade de Coimbra (HUC) and Hospital Pediátrico de Coimbra (HPC), Unidade Local de Saúde de Coimbra, the largest referral center for GEDs in Portugal. Before genetic testing, all patients had a preliminary GED diagnosis based on family history (defined as the report of a relative with either a diagnosis of an IRD and/or a pattern of vision loss coinciding with the patient's phenotype), structural and functional testing, and systemic findings. All patients were enrolled in the national GED registry (IRD-PT, retina.com.pt), which supports the aggregation of patients into families.^24^ The complete dataset until July 2025 was extracted and analyzed in this study.

### Genetic Testing

All probands were referred to the genetics department for consultation with a medical geneticist. Initial testing was performed according to clinical suspicion, comprising both traditional and next-generation approaches, and all were performed in external, certified, and accredited diagnostic laboratories. Traditional methods included Sanger Sequencing (single-gene analysis or investigation of known familial variants), multiplex ligation-dependent probe amplification (MLPA), and array-CGH. NGS panels were used in cases of phenotypes associated with a single gene or a select number of genes and in the absence of ES (Supplementary Table S1 describes the content of the gene panels). As ES became increasingly accessible, it replaced NGS panels as the primary approach. The identified genetic variants were classified according to the American College of Medical Genetics and Genomics (ACMG) standards and guidelines for the interpretation of sequence variants, from benign (1) to pathogenic (5).^25^ Pre- and post-testing genetic counseling was provided to all probands and available relatives.

### Classification of Genetic Testing Outcomes

Based on the results of genetic testing, cases were classified as solved, partially-solved, or unsolved. A case was considered solved if a likely pathogenic (LP) or pathogenic (P) variant was found in heterozygosity for autosomal dominant disease, in hemizygosity in male subjects or heterozygosity in symptomatic female subjects for X-linked disease, or in homozygosity/compound heterozygosity (two LP/P variants in trans) for autosomal recessive disease, in genes consistent with the clinical phenotype. Cases with two LP/P variants without phase information or cases with phenotype-matching (PM)-VUS, with supportive bioinformatic evidence of pathogenicity and/or positive FSA, that did not meet the ACMG criteria for reclassification) were considered partially-solved. All other cases were classified as unsolved.

### Workflow for Unsolved Cases

Cases initially classified as unsolved, defined as those in which genetic testing failed to identify a causal variant, were re-evaluated in a multidisciplinary setting to determine whether additional genetic testing was warranted. Updated NGS panels were mostly used before ES became widely accessible. When technically feasible, the evaluation was expanded to ES through realignment and reanalysis of the existing sequencing data; otherwise, a new ES test was performed. Targeted analyses included MLPA, used to detect large deletions or duplications, as well as gene-specific assays, such as investigation of known intronic variants (e.g., the c.2991+1655A>G variant in intron 26 of the *CEP290* gene) or ORF sequencing for the detection of pathogenic variants in open reading frame 15 (ORF15) region. Periodic review by the medical genetics and ophthalmology team was scheduled for cases that remained unsolved, in accordance with the Portuguese guidelines for genetic testing in IRDs.^20^

If the first test identified one or more VUS, further investigation included FSA to clarify inheritance pattern, determine whether variants within the same gene were in *cis* or *trans*, and assess whether the variant co-segregated with the disease phenotype.

In addition, a comprehensive review of published literature was performed, including internal and external case databases and public variant repositories, such as ClinVar, gnomAD, or the Leiden Open Variation Database (LOVD). Bioinformatic platforms that predict the functional impact of missense variants, such as REVEL, as well as those supporting variant interpretation, such as VarSome and Franklin, were incorporated into the analysis to aid in variant reclassification according to ACMG guidelines.^26^

### Ethics

Informed consent was obtained from all participants at the time of enrollment. The study adheres to the tenets of the Declaration of Helsinki for biomedical research and was approved by the Ethics Committee of Unidade Local de Saúde de Coimbra.^27^ All clinical and genetic data was handled in accordance with data protection regulations, and patient confidentiality was kept throughout the study.

## Results

**Figure 1.**
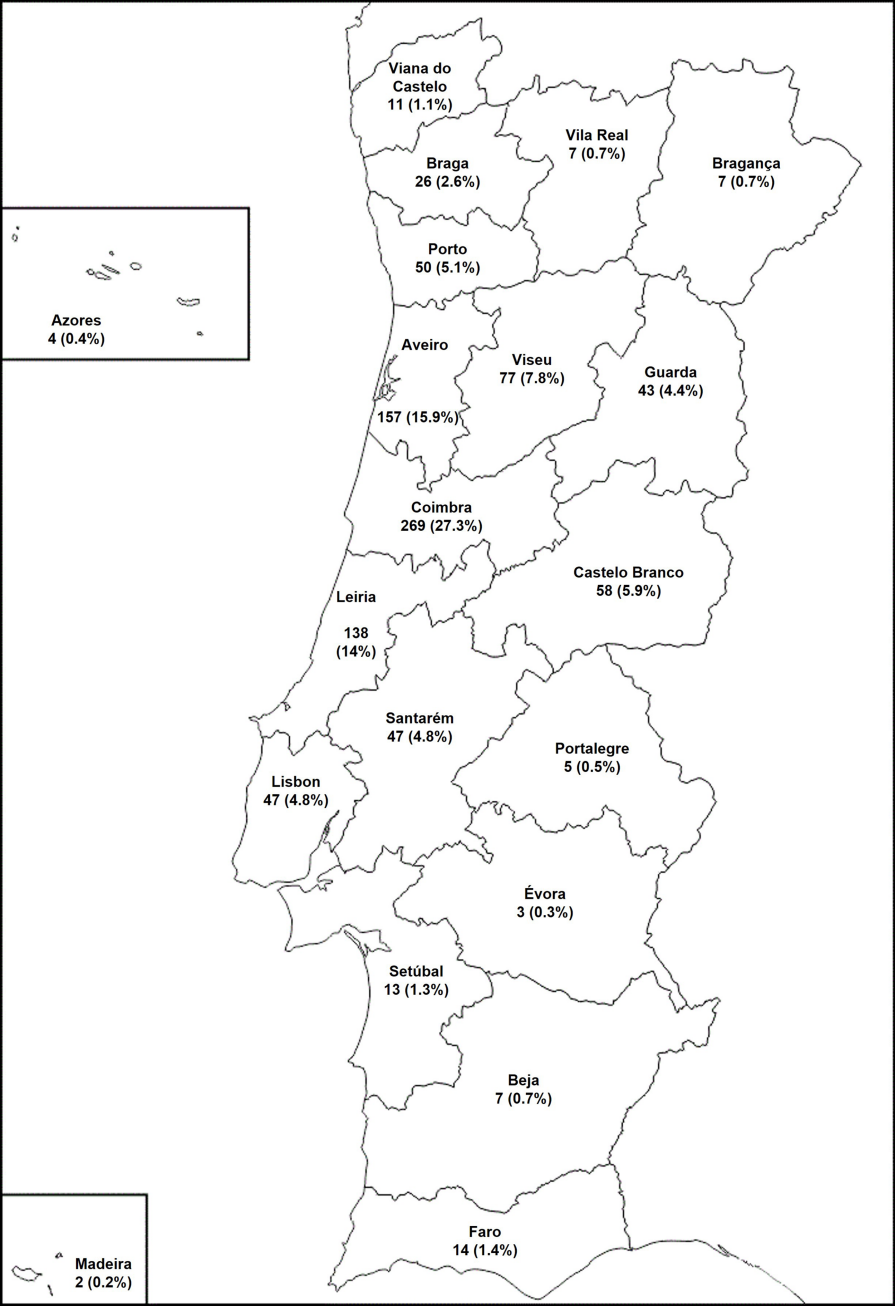
Distribution of included patients according to the district of residence.

This study included 988 patients from 800 families, identified using the national IRD-PT registry (retina.com.pt).^24^ Approximately half (53.7%, 530 cases) were men, and median age at the onset of symptoms was 15.5 years (range = 0–55 years). The first visit at our IRD referral center occurred, on average, 21.7 years later, at 39 ± 19.9 years (range = 0–86 years); molecular testing spanned from 2006 to 2025, with more than 93% of tests performed between 2018 and 2025. Mean follow up period was 90.7 ± 102.1 months. A positive family history of GED was reported in 517 patients (52.3%), and 203 (20.5%) reported a history of familial consanguinity. Figure 1 details the distribution of patients according to the district of residence.

### Solved Cases

Initial genetic testing proved successful in 475 families (594 individuals), yielding a first-diagnostic approach success rate of 59.4%. In 243 (41%) patients, an NGS panel tailored to clinical suspicion was performed, followed by ES (*n* = 146, 24.6%) and targeted testing for a known familial variant (*n* = 125, 21%). RP accounted for 179 (37.7%) of included families, followed by syndromic inherited retinal dystrophies (SIRD – *n* = 103, 21.7%), Stargardt disease (STGD; *n* = 38, 8%), and mixed cone/cone-rod dystrophies (CRDs; *n* = 25, 5.3%). Pathogenic variants involved a total of 143 different genes, of which *EYS* was the most common (*n* = 56 families, 11.8%), followed by *ABCA4* (*n* = 39, 8.2%), and *USH2A* (*n* = 31, 6.5%).

### Unsolved Cases

A total of 102 unsolved families (104 cases) were not considered eligible for further workup. Most involved negative results from either a tailored NGS panel (55 cases, 52.9%), ES (39, 37.5%), or select gene sequencing (6, 5.8%). An additional three patients (2.9%) were lost to follow-up. Clinically suspected RP still accounted for the majority of affected families (*n* = 48, 44.4%). Macular dystrophies (MDs) and CRD increased significantly in frequency when comparing to the solved group, from 4.6 to 19.2% and 5.3 to 10.6%, respectively.

### Case Reanalysis and Molecular Workup

#### Phenotype Matching Variants of Undetermined Significance

All identified phenotype matching variants of undetermined significance (PM-VUS) cases, either in homozygosity, heterozygosity, or in association with a LP or P variant were further analyzed in an attempt to support variant reclassification. In 37 families, FSA was not possible due to unavailability of family members. In seven families, FSA did not support an association between the variant and the disease and in an additional proband, an initially classified PM-VUS was reclassified as benign.

In 45 families, the combination of FSA, literature, and database review and bioinformatic analysis supported the likely association of identified PM-VUS with the clinically suspected disease; however, such data proved insufficient for variant reclassification.

For example, in F150 (P154), the NGS panel, MLPA and targeted intronic variant analysis of *ABCA4* identified a pathogenic variant (c.5882G>A, p.Gly1961Glu) and a PM-VUS (c.5196+1136del, p.?) in compound heterozygosity. This deletion is located in intron 36, where a variant associated with STGD was reported. Segregation studies supported the association of the variants with the disease. Bioinformatic prediction tools were inconclusive; as such, its classification remained unchanged.

In F195 (P223) with non-syndromic RP phenotype, a targeted NGS panel was inconclusive. Subsequent ES was negative but later reanalysis identified a homozygous variant in *IFT88* (c.1556C>T, p.Ala519Val), classified as VUS. Although variants in the *IFT88* gene are primarily associated with syndromic ciliopathies, recent data proposed the association with non-syndromic RP.^28^ Nevertheless, available data did not support reclassification.

#### Variant Reclassification

Variant reclassification was possible in 39 families (56 cases) in light of predictive tool analysis, literature review, and FSA; these PM-VUS were mostly identified by NGS panels and ES (33 families).

Two novel coding variants were identified, with sufficient evidence to support their pathogenicity. In F208 (P247/248), with a clinical diagnosis of Leber Congenital Amaurosis (LCA), ES identified the homozygous *LRAT* variant, c.149T>G (p.Val50Gly), which affects a moderately conserved residue, and further FSA allowed its classification as LP. In F211 (P254/255), and probands P269 (F225) and P270 (F226), with X-linked retinoschisis (XLRS), the hemizygous missense variant c.569T>C, p.Leu190Pro, which affects a highly conserved residue, was reported in the *RS1* gene; bioinformatic tools and FSA allowed for its classification as LP.

Likewise, in F229 (P273), an NGS panel identified variants in *HK1* and *PRPF31*. The *HK1* variant was considered not linked to the phenotype. Two *cis* variants were found in *PRPF31*, an in-frame duplication (c.1005_1007dup, p.Pro336dup), classified as a VUS (no predicted functional impact), and a partial deletion spanning from exon 10 to exon 14 and part of the 3′ UTR region – c.(?_1041)_(*287_?)del, p.? – which was reclassified as LP after FSA, yielding the molecular diagnosis of *PRPF31*-associated autosomal dominant RP.

Phenotype re-analysis may also aid in variant reclassification. For example, initial clinical evaluation of F219 (P263) led to a diagnosis of CRD. Genetic testing revealed the homozygous c.1150C>T (p.Arg384Trp) variant in the *ARSG* gene, initially classified as VUS. Its location (exon 10, the protein's sulfatase domain) was within the same domain as other LP/P variants described in this gene, classically associated with an RP phenotype, and FSAs were positive.^29^ Clinical re-evaluation supported its association with the described genotype, allowing for variant reclassification. A summary of cases that underwent variant reclassification is available in Table 1.

**Table 1.** Cases That Underwent Variant Reclassification Through Familial Studies, Literature Review, and Integration of Bioinformatic Tools

Variant Reclassification									
ID	Family	Gender	Age at Diagnosis	GED Subtype	Genetic Testing	Gene	Variants	Status	ACMG Criteria for Reclassification
P43	41	M	38	CRD	NGS panel	*CERKL*	LP - c.316C>A p.(Arg106Ser);	Homozygous	PM5m, PM2m, PM3m
							LP - c.316C>A p.(Arg106Ser)		
P44	42	M	56	RP	NGS panel	*IMPG1*	LP - c.1876C>T p.(Leu626Phe);	Homozygous	PS4st, PM2m
							LP - c.1876C>T p.(Leu626Phe)		
P208	182	M	62	RP	NGS panel	*CRB1*	LP - c.1760G>A p.(Cys587Tyr);	Homozygous	PP1s, PM2m, PP3s, PP2s, PM3s
							LP - c.1760G>A p.(Cys587Tyr)		
P212	186	M	59	RP	NGS panel	*IMPG1*	LP - c.1876C>T p.(Leu626Phe)	Heterozygous	PS4st, PM2m
P228	200	M	24	MD	Familial variant testing	*PRPH2*	LP - c.623G>A p.(Gly208Asp)	Heterozygous	PM2m, PM5m, PM1m, PP3s, PP1s
P229		M	65		NGS panel				
P230		M	58		Familial variant testing				
P231	201	M	79	MD	NGS panel	*PRPH2*	LP - c.695C>T p.(Ala232Val)	Heterozygous	PM2m, PM5m, PM1m, PP3s
P232		M	53		Familial variant testing				
P233	202	F	60	RP	Familial variant testing	*IMPG1*	LP - c.1876C>T p.(Leu626Phe);	Homozygous	PM2m, PS4s, PP1s
P234		F	62		NGS panel		LP - c.1876C>T p.(Leu626Phe)		
P235	203	M	13	RP	ES	*RPGR*	LP - c.778+5G>A p.?	Hemizygous	PM2m, PP3m, PP1s, PP4s
P236		M	15		NGS panel				
P237		F	45		Familial variant testing				
P238	204	F	10	CRD	Familial variant testing	*RAB28*	LP - c.390A>C p.(Lys130Asn);	Homozygous	PM2m, PP3m, PP1m
P239		M	16		ES		LP - c.390A>C p.(Lys130Asn)		
P240	205	M	39	SIRD	Sanger Sequencing	*ABCC6*	LP - c.1171A>G p.(Arg391Gly);	Comp heterozygous	[PM2m, PP3s, PM1s, PM3s, PP4s]
P241		F	27		Sanger Sequencing		P - c.3421C>T p.(Arg1141*)		[PM3st, PVS1vs, PM2m]
P242	206	M	75	MD	Familial variant testing	*PRPH2*	LP - c.695C>T p.(Ala232Val)	Heterozygous	PM1m, PM2m, PM5m, PP3s
P243		M	77		Familial variant testing				
P244		F	44		ES				
P245	207	F	30	CSNB	NGS panel	*RDH5*	LP - c.355G>A p.(Gly119Arg);	Homozygous	PM2m, PP3m, PP1s, PP4s
P246		M	58		Familial variant testing		LP - c.355G>A p.(Gly119Arg)		
P247	208	F	22	LCA	Familial variant testing	*LRAT*	LP - c.149T>G p.(Val50Gly);	Homozygous	PM2m, PM1s, PP1s, PM3s, PP4s
P248		F	33		ES		LP - c.149T>G p.(Val50Gly)		
P249	209	M	9	RP	NGS panel	*IMPDH1*	LP - c.848A>T p.(Asn283IIe)	Heterozygous	PM2m, PM5s, PP3m, PP1s
P250		F	41		Familial variant testing				
P251		M	11		ES				
P252	210	M	9	RP	Familial variant testing	*PRPF31*	LP - c.1A>T p.?	Heterozygous	PS4m, PS1st, PVS1m, PM2m, PP1s
P253		M	28		NGS panel				
P254	211	M	15	XLRS	Familial variant testing	*RS1*	LP - c.569T>C p.(Leu190Pro)	Hemizygous	PM1m, PP2s, PM2m, PP3m, PP1s, PP4s
P255		M	61		Sanger Sequencing				
P256	212	F	31	RP	NGS panel	*WDR19*	LP - c.2782A>T p.(Ile928Phe);	Comp heterozygous	[PM2m, PP3m, PM3m];
							P - c.3112C>T p.(Arg1038*)		[PM3st, PVS1vs, PM2m]
P257	213	F	55	RP	ES	*USH2A*	P - c.10182G>A p.(Lys3394=);	Comp heterozygous	[PM2m, PP3s, PS3st, PM3m];
							P - c.907C>A p.(Arg303Ser)		[PM3st, PM2m, PM5m, PM1m]
P258	214	M	28	MD	ES	*RDH12*	LP - c.701G>A p.(Arg234His);	Comp heterozygous	[PS3s, PM1m, PP2s, PM2m];
							P - c.381del p.(Val128*)		[PVS1vs, PM2m]
P259	215	F	38	STGD	NGS panel	*ABCA4*	LP - c.3113C>T p.(Ala1038Val);	Comp heterozygous	[PM1m, PP2s, PM2m, PP5s];
							LP - c.3386G>T p.(Arg1129Leu)		[PS3s, PM1m, PP2s, PM2m, PM5m, PP3s]
P260	216	M	54	SIRD	NGS panel	*ARSG*	LP - c.1326del p.(Ser443Alafs*12);	Homozygous	PS2s, PVS1st, PM2m
							LP - c.1326del p.(Ser443Alafs*12)		
P261	217	M	16	CRD	NGS panel	*RAB28*	LP c.430C>T p.(His144Tyr);	Comp heterozygous	[PP1m, PM3s, PM2m, PP3m];
							LP c.(?_-1)_(75+1_392-1)del		[2C, 4L]
P262	218	F	28	RP	NGS panel	*TOPORS*	LP - c.2550_2553del p.(Asp850Glufs*15)	Heterozygous	PS4m, PVS1st, PM2m
P263	219	F	36	SIRD	ES	*ARSG*	LP - c.1150C>T p.(Arg384Trp);	Homozygous	PP1m, PP4m, PM2m
							LP - c.1150C>T p.(Arg384Trp)		
P264	220	F	41	CRD	NGS panel	*RDH12*	LP - c.701G>A p.(Arg234His);	Comp heterozygous	[PS3s, PM1m, PP2s, PM2m];
							LP - c.464C>T p.(Thr155Ile)		[PS4s, PS3s, PM2m, PP3m]
P265	221	M	17	CRD	NGS panel	*CERKL*	LP - c.497C>T p.(Pro166Leu);	Comp heterozygous	[PM2m, PP5s, PM3m, PP1s];
							P - c.847C>T p.(Arg283*)		[PM3vs, PP1s, PVS1vs, PM2m]
P266	222	F	42	RP	ES	*CERKL*	LP - c.356G>A p.(Gly119Asp);	Comp heterozygous	[PM3vs, PM2m];
							P - c.847C>T p.(Arg283*)		[PM3vs, PP1s, PVS1vs, PM2m]
P267	223	M	44	MD	Sanger Sequencing	*BEST1*	LP - c.851A>G p.(Tyr284Cys)	Heterozygous	PP1s, PM1m, PP2s, PP2m, PM5m, PP3m, PP4s
P268	224	M	42	MD	NGS panel	*BEST1*	LP - c.304T>C p.(Trp102Arg)	Heterozygous	PS4st, PM1m, PM2m
P269	225	M	12	XLRS	Sanger Sequencing	*RS1*	LP - c.569T>C p.(Leu190Pro)	Hemizygous	PM1m, PP2s, PM2m, PP3m, PP1s
P270	226	M	35	XLRS	Sanger Sequencing	*RS1*	LP - c.569T>C p.(Leu190Pro)	Hemizygous	PM1m, PP2s, PM2m, PP3m, PP1s
P271	227	M	39	CRD	ES	*DRAM2*	LP - c.517+5C>A p.(?);	Homozygous	PS3m, PM3m, PP1m, PM2m
							LP - c.517+5C>A p.(?)		
P272	228	M	62	CRD	NGS panel	*EYS*	LP - c.9059T>C p.(Ile3020Thr);	Comp heterozygous	[PM3s, PM2m, PM1s, PP4s];
							P - c.4120C>T p.(Arg1374*)		[PM3vs, PVS1vs, PM2m]
P273	229	F	43	RP	NGS panel	*PRPF31*	LP - c.(?_1041)_(*287_?)del	Heterozygous	2B
P274	230	F	78	RP	NGS panel	*PRPF31*	LP - c.855+5G>A p.?	Heterozygous	PS4m, PP1s, PM2m, PP3s
P275	231	F	23	RP	NGS panel	*LRAT*	LP - c.496dup p.(Thr166Asnfs*18);	Comp heterozygous	[PVS1st, PM2m];
							LP - c.163C>G p.(Arg55Gly)		[PM3st, PM2m, PM5m, PM1s]
P276	232	M	19	CRD	NGS panel	*PPT1*	LP - c.583T>C p.(Tyr195His);	Comp heterozygous	[PP3st, PM2m, PS3st];
							LP - c.541G>A p.(Val181Met)		[PS3s,PM1m, PM2m]
P277	233	M	6	STGD	NGS panel	*ABCA4*	LP - c.5196+1056A>G p.?;	Comp heterozygous	[PM3vs, PM2m];
P278		F	4		Familial variant testing		P - c.[1622T>C,4328G>A] p.[Leu541Pro,Arg1443His]		[(PM3vs, PP1s, PS3s, PP3st, PM2m, PP2s)(PM3vs, PS3s, PM1m, PP2s, PM2m, PM5m, PP3s)]
P279	234	M	51	MD	Sanger Sequencing	*CDHR1*	LP - c.783G>A p.(Pro261=);	Homozygous	PP1s, PM2m, PP3m
							LP - c.783G>A p.(Pro261=)		

CRD, cone-rod dystrophy; CSNB, congenital stationary night blindness; ES, exome sequencing; FH, foveal hypoplasia; GED, genetic eye disorders; ID, identifier; LCA, Leber congenital amaurosis; LP, likely pathogenic; m, moderate; MD, macular dystrophy; NGS panel, next-generation sequencing panel; P, pathogenic; RP, retinitis pigmentosa; s, supporting; SIRD, syndromic inherited retinal dystrophy; st, strong; STGD, Stargardt disease; vs, very strong; XLRS, X-linked retinoschisis.

#### Re-Evaluation by NGS Panels, ES, and ES-Reanalysis

NGS panels, ES, and ES reanalysis proved unsuccessful in identifying P, LP, or even PM-VUS in a total of 34 families (34 probands). These strategies were commonly used after a negative NGS panel (17), ES (8), or targeted gene sequencing (4). Six probands underwent at least three negative testing strategies. Median time to re-evaluation was 3.25 years (interquartile range [IQR] = 2.00 years).

This re-testing strategy proved successful in 25 families, 11 through an NGS panel, and 14 through ES or ES reanalysis. For example, in F285 (P335), an initial NGS panel was negative; ES revealed a 198 kb deletion in chromosome 6 (6q23.3), partially including the *AHI1* gene, which was further confirmed by arrayCGH, and the c.3196C>T (p.Arg1066*) variant, in hemizygosity due to the deletion, classified as LP. Although biallelic *AHI1* variants are classically associated with Joubert syndrome, there are reported instances of non-syndromic RP.^30^ As systemic evaluation was negative, a molecular diagnosis of non-syndromic RP associated with the *AHI1* gene was established.

Interestingly, in F252 (P302), with syndromic-RP, initial ES identified a VUS, deemed not relevant. Follow-up mitochondrial genome analysis by NGS identified the P variant m.8993T>G in heteroplasmy (95%) in the *MT*-*ATP6* gene, classified as pathogenic in the Mitochondrial DNA Mutation and Polymorphism database; this confirmed the diagnosis of RP associated with the Neuropathy, Ataxia, and Retinitis Pigmentosa/Maternally Inherited Leigh Syndrome (NARD/MILS) spectrum.^31^

In F286 (P336), initial genetic testing identified a single heterozygous LP variant in *ABCA4*. Subsequent ES revealed the presence of an additional heterozygous variant, the hypomorphic c.5603A>T (p.Asn1868Ile), which had not been detected previously. FSA confirmed that the two variants are in trans, thus providing the molecular diagnosis.

#### Evaluation of Exon-Level Deletions/Dupli-cations, Intronic Variants, and Highly Repetitive and GC-Rich Regions

Evaluation of exon-level deletions/duplications, intronic variants, and investigation of the highly repetitive and GC-rich region of the ORF15 exon of the *RPGR* gene was performed in 28 families. In four families (F302-305, P352-370), causal variants in the ORF15 region were detected and allowed for a molecular diagnosis. Exon level deletion/duplication with MLPA was also successful in an additional 12 families, 5 linked to *EYS* and 3 to the *ABCC6* genes. Evaluation of the intron 26 of the *CEP290* gene in F66 (P68) and F314 (P381) identified the P non-coding variant c.2991+1655A>G (p.Cys998X) in compound heterozygosity and homozygosity, respectively, which leads to the creation of a cryptic splice donor site.

Remarkably, in F311 (P378), an NGS panel failed to identify any pathogenic variants. However, no coverage for exons 11 to 51 of *COL4A5* was possible, suggesting a possible large hemizygous deletion; MLPA identified the c.(609+1_610–1)_(998?) deletion, ranging from exons 11 to 51 of the *COL4A5* gene. This deletion had not been previously reported, although larger overlapping deletions were described in association with Alport syndrome, allowing its classification as LP.^32^ A summary of cases that underwent successful reanalysis is available in Table 2.

**Table 2.** Solved Cases Through Additional Next Generation Sequencing (NGS) Panels, Exome Sequencing (ES), ORF15 Sequencing, Muliplex Ligation-Dependent Probe (MPLA), and Intronic Variant Testing

Solved Cases Through Additional NGS Panels, ES, ORF15 Sequencing, MLPA, and Intronic Variants Evaluation									
ID	Family	Gender	Age at Diagnosis	GED Subtype	First Test	Last Test	Gene	Variants	Status
P291	246	F	62	HON	Familial variant testing	—	*OPA1*	LP - c.1560_1562del p.(Glu521del)	Heterozygous
P292		M	24		NGS panel	NGS panel			
P293		F	55		Familial variant testing	—			
P294		F	55		Familial variant testing	—			
P295		F	84		Familial variant testing	—			
P296		F	29		Familial variant testing	—			
P297	247	F	4	FH	Sanger Sequencing	NGS panel	*HPS6*	P - c.223C>T p.(Gln75*);	Homozygous
								P - c.223C>T p.(Gln75*)	
P298	248	F	26	SIRD	Sanger Sequencing	NGS panel	*USH2A*	P - c.7932G>A p.(Trp2644*);	Homozygous
								P - c.7932G>A p.(Trp2644*)	
P299	249	M	48	LCA	Sanger Sequencing	NGS panel	*RPE65*	LP - c.1583G>T p.(Gly528Val);	Homozygous
								LP - c.1583G>T p.(Gly528Val)	
P300	250	M	0	FH	ArrayCGH	NGS panel	*PAX6*	P - c.781C>T p.(Arg261*)	Heterozygous
P301	251	M	29	LCA	Sanger Sequencing	NGS panel	*RPGRIP1*	LP - c.(1151+1_1152-1)_(3099+1_3100-1)del;	Homozygous
								LP - c.(1151+1_1152-1)_(3099+1_3100-1)del	
P302	252	F	5	SIRD	ES	NGS panel	*MT-ATP6*	P - m.8993T>G; heteroplasmy (95%)	Heteroplasmy
P303	253	F	24	RP	NGS panel	NGS panel	*EYS*	P - c.(2023+1_2024-1)_(2259+1_2260-1)del;	Homozygous
								P - c.(2023+1_2024-1)_(2259+1_2260-1)del	
P304	254	M	39	SIRD	ArrayCGH	NGS panel	*USH1G*	LP - c.183T>A (p.Cys61*);	Homozygous
								LP - c.183T>A (p.Cys61*)	
P305	255	M	27	SIRD	Sanger Sequencing	NGS panel	*USH2A*	LP - c.(7300+4_7301-1)_(9371+1_9372-1)del;	Homozygous
								LP - c.(7300+4_7301-1)_(9371+1_9372-1)del	
P306	256	M	3	CSNB	NGS panel	NGS panel	*CACNA1F*	P - c.952_954del p.(Phe318del)	Hemizygous
P17	15	M	57	FH	NGS panel	ES	*HPS5*	P - chr11:18301399-18307009 (exon 20-23 del);	Homozygous
								P - chr11:18301399-18307009 (exon 20-23 del)	
P58	56	M	13	CRD	ES	ES	*OPN1LW/OPN1MW*	P - chrX:153418469-153455711 (exon 4 OPN1LW - exon 3 OPN1MW del)	Hemizygous
P71	F69	M	54	MD	NGS panel	ES	*PRPH2*	P - chr6:42689492-42690072 (exon 1 del)	Heterozygous
P330	280	M	30	HON	Sanger Sequencing	ES	*KIF1A*	LP - c.914C>T p.(Pro305Leu)	Heterozygous
P331	281	M	65	RP	Sanger Sequencing	ES	*EYS*	P - c.(2023 1_2024-1)_(2259 1_2260-1)del;	Homozygous
								P - c.(2023 1_2024-1)_(2259 1_2260-1)del	
P332	282	F	26	HON	NGS panel	ES	*MT-ND4*	P - m.11778G>A p.Arg340His	Homoplasmy
P333	283	M	49	RP	ES	ES	*CNGB1*	LP - c.2285G>T p.(Arg762Leu);	Comp heterozygous
								P - c.1958-1G>A p.?	
P334	284	M	16	SIRD	Sanger Sequencing	ES	*MYO7A*	LP - c.5510T>A p.(Leu1837His);	Comp heterozygous
								LP - c.6026C>A p.(Ala2009Asp)	
P335	285	F	22	RP	NGS panel	ES	*AHI1*	LP - del. 6q23.3(135632761_135830306;	Hemizygous
								LP - c.3196C>T p.(Arg1066*)	
P336	286	M	50	MD	NGS panel	ES	*ABCA4*	LP - c.4919G>A p.(Arg1640Gln);	Comp heterozygous
								LP - c.5603A>T (Asn1868lle)	
P337	287	F	40	RP	NGS panel	ES	*CERKL*	P - c.847C>Tp.(Arg283*);	Homozygous
								P - c.847C>Tp.(Arg283*),	
P338	288	M	39	RP	NGS panel	ES	*AGBL5*	LP - c.943T>G p.(Tyr315Asp);	Homozygous
								LP - c.943T>G p.(Tyr315Asp)	
P339	289	M	42	CRD	Sanger Sequencing	ES	*DRAM2*	LP - c.517+5C>A p.(?)	Homozygous
								LP - c.517+5C>A p.(?)	
P340	290	F	49	SIRD	NGS panel	ES	*CEP250*	LP - c.4006C>T p.(Arg1336*);	Homozygous
								LP - c.4006C>T p.(Arg1336*)	
P352	302	F	48	RP	Familial variant testing	—	*RPGR*	P - c.2763_2764del p.(Glu922Glyfs*156)	Hemizygous
P353		M	10		Familial variant testing	—			
P354		M	33		Familial variant testing	—			
P355		F	12		Familial variant testing	—			
P356		F	40		Familial variant testing	—			
P357		F	22		Familial variant testing	—			
P358		M	32		ES	ORF15			
P359	303	M	25	RP	ES	ORF15	*RPGR*	LP - c.2872del p.(Glu958Lysfs*131)	Hemizygous
P360		M	24		NGS panel	ORF15			
P361		F	51		Familial variant testing	—			
P362		M	29		Familial variant testing	—			
P363		F	55		Familial variant testing	—			
P364		F	53		Familial variant testing	—			
P365		M	23		NGS panel	ORF15			
P366	304	M	49	RP	Familial variant testing	—	*RPGR*	P - c.2426_2427del p.(Glu809Glyfs*25)	Hemizygous
P367		M	29		ES	ORF15			
P368	305	M	18	RP	NGS panel	ORF15	*RPGR*	P - c.2501del p.(Glu834Glyfs*255)	Hemizygous
P369		F	42		Familial variant testing	—			
P370		M	16		Familial variant testing	—			
P68	66	F	62	RP	NGS panel	Intronic variant testing	*CEP290*	P - c.2991+1655A>G, p.(?);	Comp heterozygous
								P - c.6271-8T>G p.(?)	
P371	306	M	65	RP	Familial variant testing	—	*EYS*	LP - c.2225del p.(Cys742Leufs*36);	Comp heterozygous
P372		M	56		NGS panel	MPLA		P - c.(2023+1_2024-1)_(2259+1_2260-1)del	
P373	307	M	27	RP	ES	MPLA	*EYS*	P - c.(2023+1_2024-1)_(2259+1_2260-1)del;	Comp heterozygous
P374		F	42		Familial variant testing	—		P - c.5928-2A>G p.?	
P375	308	F	38	SIRD	NGS panel	MPLA	*USH2A*	LP - c.(7300+1_7301-1)_(9371+1_9372-1)del;	Homozygous
								LP - c.(7300+1_7301-1)_(9371+1_9372-1)del	
P376	309	F	30	RP	NGS panel	MPLA	*EYS*	LP - c.8834G>A p(Gly2945Glu);	Comp heterozygous
								P - c.(2023+1_2024-1)_(2259+1_2260-1)del	
P377	310	M	74	SIRD	Sanger Sequencing	MPLA	*ABCC6*	P - c.(2995+1_2996-1)_(3883-66_4209-1)del	Heterozygous
P378	311	M	23	SIRD	NGS panel	MPLA	*COL4A5*	LP - c.(609+1_610-1)_(*998_?)del	Hemizygous
P379	312	M	54	SIRD	Sanger Sequencing	MPLA	*ABCC6*	P - del2-30 p.0?;	Homozygous
								P - del23-29 p.0?	
P380	313	M	31	RP	NGS panel	MPLA	*EYS*	P - c.4120C>T p.(Arg1374);	Comp heterozygous
								P - c.2024-?_c.2259+?del p.(?)	
P381	314	M	0	LCA	NGS panel	Intronic variant testing	*CEP290*	P - c.2991+1655A>G (p.Cys998X);	Homozygous
								P - c.2991+1655A>G (p.Cys998X)	
P382	315	F	1	FH	NGS panel	MPLA	*OCA2*	P - c.1327G>A (p.Val443Ile);	Comp heterozygous
								P - deletion of intron 6 and exon 7	
P383	316	F	64	SIRD	Familial variant testing	—	*ABCC6*	LP - c.1703T>C p.(Phe568Ser);	Comp heterozygous
								P - c.(?_37-1)_(*1_?)del	
P384		M	57		Sanger Sequencing	MPLA			
P385	317	F	42	SIRD	Sanger Sequencing	MPLA	*ABCC6*	P - deletion of exons 2-30;	Comp heterozygous
								P - deletion of exons 23-29	
P386	318	M	42	RP	ES	MPLA	*EYS*	P - c.(862+1_863-1)_(1056+1_1184+407)del;	Comp heterozygous
								P - c.4120C>T p.(Arg1374)	

HON, hereditary optic neuropathy; MLPA, multiplex ligation-dependent probe amplification; ORF15, *RPGR* open reading frame 15.

#### Additional Relevant Cases

In F310 (P377) and F322 (P390), with biopsy proven pseudoxanthoma elasticum (PXE), sequencing and MLPA of *ABCC6* identified only one pathogenic variant [c.(2995+1_2996-1)_(3883-66_4209-1)del and c.3421C>T(p.Arg1141*), respectively] in heterozygosity, in the *ABCC6* gene. Although *ABCC6*-related PXE is classically associated with biallelic mutations, recent reports have described *forme-fruste* phenotypes in heterozygous carriers.^33^ Given the positive histology, such rare presentation was assumed in these two cases (Supplementary Fig. S1 shows the widefield color fundus photographs of P377).

A novel candidate gene was identified in F321 (P389). An ES panel revealed a homozygous frameshift variant c.618_618+11del (p.Phe207fs) in the *SLC66A1* gene, classically classified as of uncertain significance; reports have recently supported its pathogenicity in autosomal recessive RP, which allowed for a molecular diagnosis.^34^^,^^35^ Emerging data on gene-phenotype associations allowed for the molecular diagnosis of F214 (P258), in which ES identified compound heterozygous variants in *RDH12*: the frameshift variant c.381del p.(Val128), classified as pathogenic, and the missense variant c.701G>A p.(Arg234His), described as a hypomorphic allele. While *RDH12* was classically linked to LCA/early-onset severe retinal dystrophy, recent data has broadened its phenotypic spectrum to include RP and MD. In fact, pathogenic variants in the *RDH12* gene in *trans* with the c.701G>A hypomorphic allele have been linked to retinopathy with macular involvement, allowing for the molecular diagnosis in this patient.^36^

### Diagnostic Yield

**Figure 2.**
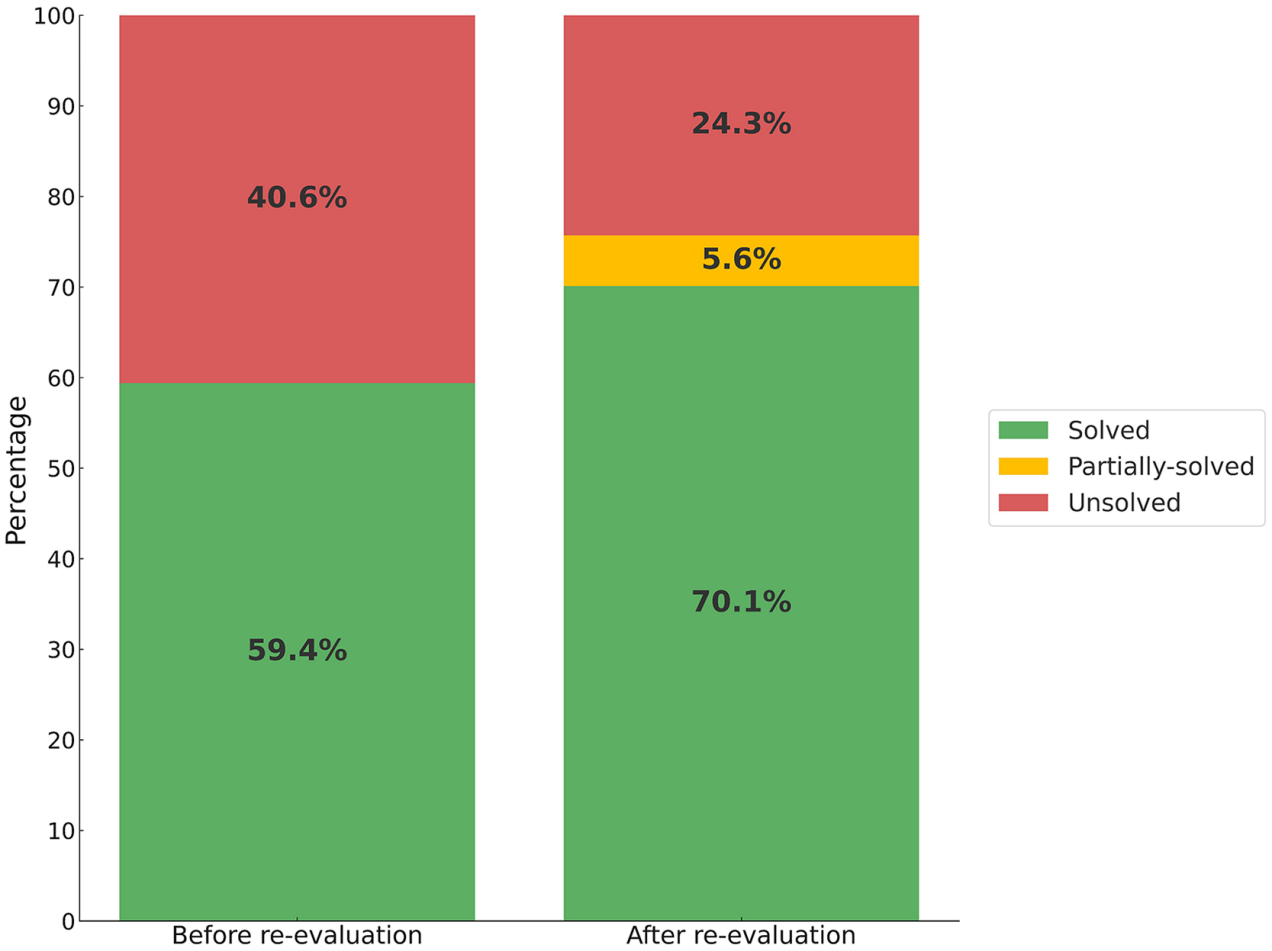
Diagnostic yield of the entire cohort before and after case-by-case re-evaluation.

**Figure 3.**
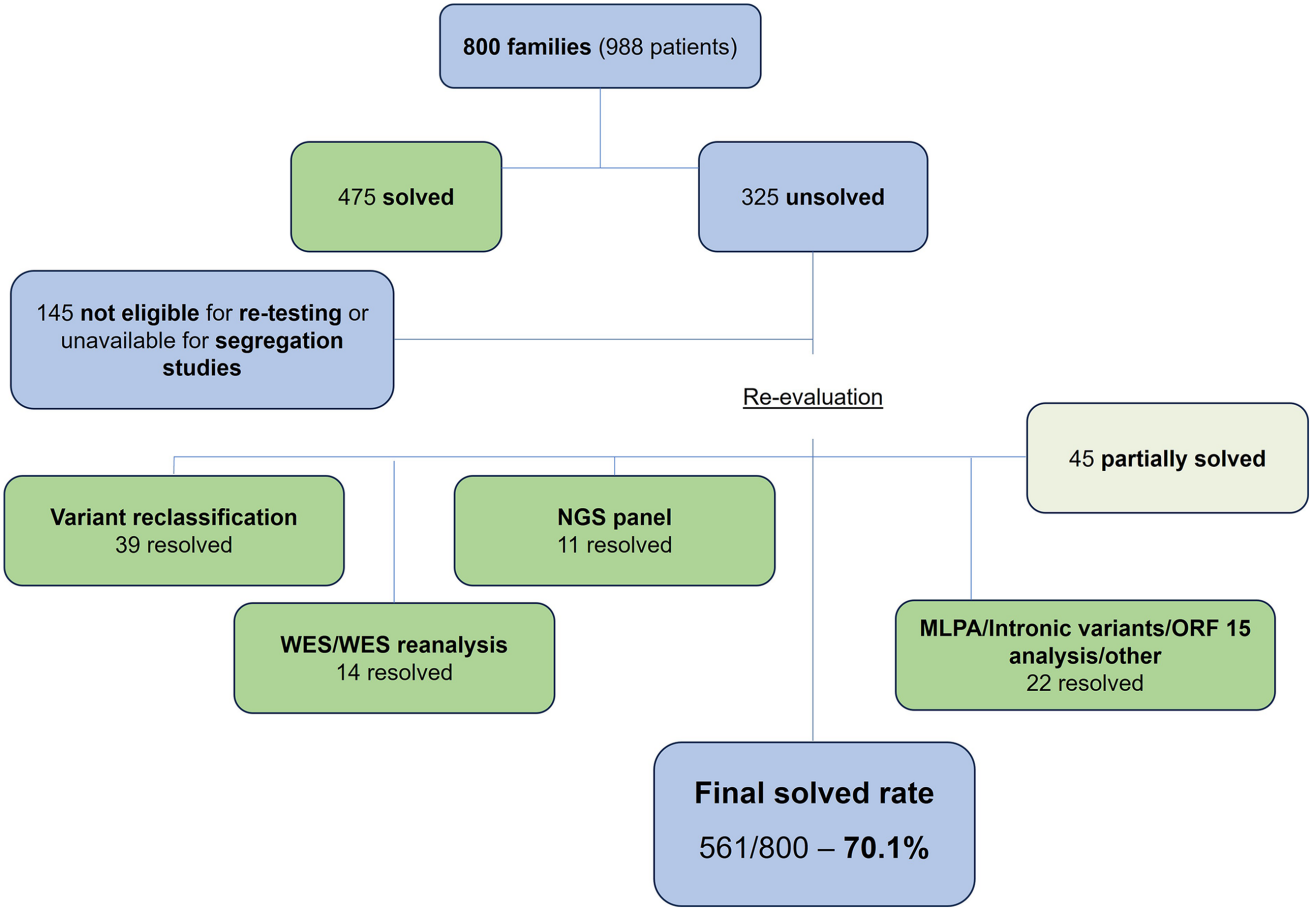
Re-evaluation strategy workflow for the entire cohort. MLPA, multiplex ligation-dependent probe amplification; NGS, next generation sequencing; ORF, open reading frame; WES, whole exome sequencing.

Overall, variant reclassification (39 families), NGS panels (11), ES/ES reanalysis (14), and other strategies (22), such as ORF15 exon sequencing, MLPA analysis, and evaluation of intronic variants, allowed for an improvement of the familial molecular diagnostic rate of 10.7%, from 59.4% (475) to 70.1% (561 families). An additional 45 (5.6%) were considered partially-solved – Figure 2. Figure 3 depicts the case reanalysis workflow for the entire cohort. The distribution of IRD subtypes and associated genes per resolution status is available in Table 3. The results of the genetic testing for all partially-solved and solved individuals are provided in Supplementary Table S2.

**Table 3.** Clinical Phenotype, Genotype, and Molecular Diagnosis Characterization by Case Resolution Status, Along With Number of Families That Underwent Reclassification

Genetic Landscape						
GED Subtype	Gene (Families)	Solved	Partially- Solved	Unsolved	Total	Variant Reclassification, Per Gene (Families)
ACHM	*CNGB3* (9); *CNGA3* (3); *ATF6* (1); *PDE6C* (1)	14 (100%)	—	—	14	
CD	*CYP4V2* (6); *CHM* (4); *C1QTNF5* (1); *OAT* (1)	8 (57.1%)	2 (14.3%)	4 (28.6%)	14	
CRD	*KCNV2* (10); *PROM1* (4); *RPGR* (4); *ABCA4* (3); *CER*KL (4); *DRAM2* (2); *EYS* (2); MFSD8 (2); *RAB28* (2); *RDH12* (2); *BBS1* (1); *CACNA1F* (1); *CACNA2D4* (1); *CHDR1* (1); *GUCA1A* (1); *GUCY2D* (1); KIZ (1); *OPN1LW*/*OPN1MW* (1); *POC1B* (1); *PPT1* (1); *TTLL5* (1)	36 (53.7%)	5 (7.5%)	26 (38.8%)	67	*CERKL* (2); *DRAM2* (1); *EYS* (1); *PPT1* (1); *RAB28* (2); *RDH12* (1)
CSNB	*RDH5* (7); *CACNA1F* (3); *GRK1* (1); *SLC24A1* (1); *TRPM1* (1)	10 (71.4%)	1 (7.1%)	3 (21.4%)	14	*RDH5* (1)
FH	*PAX6* (10); *TYR* (6); *SLC38A8* (3); *OCA2* (2); *GPR143* (1); *HPS5* (1); *HPS6* (1); *MITF* (1); *RARB* (1); *SLC24A5* (1)	23 (65.7%)	—	12 (34.3%)	35	
HON	*OPA1* (11); *MT*-*ND4* (4); *WFS1* (2); *FAHN* (1); *KIF1A* (1); *MT*-*DN6* (1)	17 (77.3%)	3 (13.6%)	2 (9.1%)	22	
LCA	*RPE65* (11); *RPGRIP1* (4); *NMNAT1* (3); *CEP290* (2); *SPATA7* (2); *AIPL1* (1); *CRB1* (1); *GUCY2D* (1); *LCA5* (1); *LRAT* (1); *RBP4* (1)	28 (87.5%)	—	4 (12.5%)	32	*LRAT* (1)
MD	*PRPH2* (17); *BEST1* (10); *CRB1* (2); *IMPG1* (2); *IMPG2* (2); *ABCA4* (1); *CDHR1* (1); *PAX6* (1); *PRPF4* (1); *RDH12* (1); *WFS1* (1)	31 (52.5%)	2 (3.4%)	26 (44.1%)	59	*BEST1* (2); *CDHR1* (1); *PRPH2* (3); *RDH12* (1)
MIT	*MT*-*TL1* (2); *MT*-*4977* (1)	4 (80%)	—	1 (20%)	5	
RP	*EYS* (67); *RPGR* (19); *USH2A* (20); *RHO* (12); *CNGB1* (11); *CERKL* (10); *PRPF31* (9); *NR2E3* (8); *RPE65* (8); *PROM1* (7); *IMPG1* (7); *IMPG2* (6); *MERTK* (6); *CRB1* (5); *PDE6A* (5); *CEP290* (4); *PCARE* (4); *RDH12* (4); *WDR19* (4); *AGBL5* (3); *HGSNAT* (3); *PDE6B* (3); *PRPF8* (3); *AHI1* (2); *BBS2* (2); *LRAT* (2); *RDH5* (2); *RP1* (2); *RP1L1* (2); *TOPORS* (2); *ARL2BP* (1); *CDHR1* (1); *CFAP410* (1); *DNAJC21* (1); *IFT140* (1); *IMPDH1* (1); *KLHL7* (1); *MAK* (1); *MMACHC* (1); *OTX2* (1); *PEX1* (1); *RP2* (1); *SLC66A1* (1); *TULP1* (1)	209 (64.1%)	18 (5.5%)	99 (30.4%)	326	*CERKL* (1); *CRB1* (1); *IMPDH1* (1); *IMPG1* (3); *LRAT* (1); *PRPF31* (3); *RPGR* (1); *TOPORS* (1); *USH2A* (1); *WDR19* (1)
SIRD	*USH2A* (26); *ABCC6* (23); *MYO7A* (16); *BBS1* (12); *ADGRV1* (8); *ARSG* (5); *COL18A1* (5); *COL4A5* (4); *ABHD12* (3); *BBS10* (3); *CDH23* (3); *PANK2* (3); *SDCCAG8* (3); *CEP250* (2); *CNNM4* (2); *MFRP* (2); *MT*-*TL1* (2); *CDH3* (1); *COL4A4* (1); *IQCB1* (1); *MKKS* (1); *MT-ATP6* (1); *NPHP1* (1); *PCDH15* (1); *SCAPER* (1); *TTC8* (1); *USH1G* (1); *WDR19* (1)	119 (81.5%)	10 (6.8%)	17 (11.6%)	146	*ARSG* (2); *ABCC6* (1)
STGD	*ABCA4* (46)	40 (81.6%)	3 (6.1%)	6 (12.2%)	49	*ABCA4* (2)
VRP	*COL2A1* (3); *COL11A1* (1)	—	5 (83.3%)	1 (16.7%)	6	
XLRS	*RS1* (17)	17 (100%)	—	—	17	*RS1* (3)

ACHM, achromatopsia; CD, chorioretinal dystrophy; MIT, mitochondrial disorder; VRP, vitreoretinopathy.

## Discussion

Although individually rare, IRDs and HONs represent the most common cause of visual impairment in young adults in developed countries, posing a significant burden on global healthcare systems worldwide.^6^^,^^37^^–^^39^ While genetic testing, crucial for the counseling of patients and at-risk relatives, is hindered by the heterogeneity of causal genes and inheritance patterns, there is scarce available data on how a tailored, evidence-based strategy can increase its yield.^40^^,^^41^ In this report, we describe how such a personalized approach to genetic testing, with multidisciplinary genotype-phenotype analysis, integration of in silico tools, case-by-case literature review, and periodic re-evaluation allowed for causal variant identification, clarification of VUS, and an increased solved rate in the largest referral center for GEDs in Portugal.

In fact, despite the rapid development of sequencing technology, which transitioned from single-gene Sanger analysis to high-throughput, cost-effective broad genome approaches, a significant proportion of patients are still left without a definite diagnosis.^42^^,^^43^ A recent meta-analysis which pooled data from 105 studies spawning over 11 years (2011–2022) showed that the percentage of solved cases with NGS methods was 61.3% overall, with the lowest rate (57.7%) reported in CRD; in publications from 2017 onward, this rate rises to 62.8%, reflecting broader panel coverage, and, critically, increased knowledge on gene-disease associations and variant pathogenicity. ES demonstrated a higher diagnostic yield (73.5%) compared with targeted NGS panels (59.7%), with the benefit of not requiring a deep knowledge of the spectrum of causal variants, allowing for later reanalysis as more data become available.^8^^,^^44^

In our study, initial genetic testing provided the diagnosis in 475 families; when excluding individuals tested for known familial variants, 65.5% of cases were evaluated through either an NGS (243 patients) or an ES (146 patients) assay, highlighting the increased adoption of these newer methodologies. Further retesting provided the diagnosis in an additional 25 families. An NGS panel resolved a previously unsolved case (P297) of foveal hypoplasia in a 4-year-old female, identifying a homozygous P *HPS6* variant (c.223C>T, p.Gln75*) consistent with Hermansky-Pudlak syndrome. Although no specific gene therapies are yet available, the molecular diagnosis enables clinicians to properly anticipate and manage potential life-threatening systemic complications.^45^ ES allowed for the detection of a 198kb deletion in chromosome 6 (6q23.3), partially including the *AHI1* gene, or the hypomorphic variant c.5603A>T (p.Asn1868Ile), in the *ABCA4* gene, both overlooked by NGS panels. The latter hypomorphic variant, which was previously considered benign due to its high frequency in the general population, might explain 50% of unsolved *ABCA4*-related disease cases, when in *trans* with a pathogenic variant; an additional 3 patients with *ABCA4*-related disease carried this hypomorphic variant in our cohort.^46^ Given that an estimated 250 new gene-disease associations and 9200 new variant-disease associations are reported every year, both the ACMG and the Portuguese Society of Ophthalmology and Portuguese Society of Human Genetics Joint Clinical Practice Guidelines recommend case and variant-level reanalysis every 2 years.^20^^,^^47^ Although this strategy bears costs, which cannot be overlooked in a real-world scenario, patients with a negative result in our cohort were retested at a median of 3.25 years, which is in line with current recommendations.

Detection of CNVs with NGS panels remains a challenge, and although ES has progressively evolved to successfully detect most CNVs, earlier assays presented limitations.^16^^,^^48^ In these cases, MLPA can provide additional diagnostic yield.^49^^,^^50^ In our cohort, this enabled the identification of previous overlooked LP or P variants in the *USH2A*, *EYS*, *COL4A5*, *OCA2*, and *ABCC6* genes. Furthermore, most conventional NGS panels and ES exhibit insufficient coverage of repetitive/GC-rich sequences, such as ORF15 of the *RPGR* gene. Sequencing of the ORF15 exon has been validated as an efficient strategy in the diagnosis of X-linked RP, and provided the diagnosis in an additional 4 families in our cohort.^51^ Additionally, whereas modern ES kits have high rates of deep intronic variant detection, single variant testing remains a useful tool in cases of negative NGS panels. For example, targeted sequencing of the c.2991+1655A>G, the most prevalent LCA-associated *CEP290* allele, allowed the molecular diagnosis in F66 and F314 – RP/LCA phenotype, respectively; in F242, 262, and 295, with the same phenotype and negative NGS panel/ES assay, the same strategy proved unsuccessful.^52^^,^^53^ In fact, the reported diagnostic yield is estimated to be approximately 55% to 75%, reflecting the role of unknown variants or gene-disease associations that may account for a significant proportion of patients with LCA.^54^^,^^55^

Even though advanced molecular testing protocols enable the identification of pathogenic variants, they also frequently reveal VUS, whose significance is challenging to interpretate. Our report illustrates how clinical and functional data aids the reclassification of VUS as LP or P, thereby allowing 39 families to be solved. On the other hand, VUS identified in eight families were considered not associated with the phenotype, therefore avoiding potential errors in clinical management or genetic counseling. This is a labor-intensive task, with one study estimating an average 105 minutes required per analysis, and for which no predictors of success have been described to date.^56^ This was evident for F195 (likely *IFT88-*associated non-syndromic RP) – and F321 (*SLC66A1*-associated autosomal recessive RP) for whom novel gene-disease associations could only be established after a comprehensive literature review and case discussion.

Along with genotype-phenotype analysis, extensive literature review and integration of in silico tools, a well-curated internal database is critical for accurately classifying novel variants, as was the case of the c.569T>C in the *RS1* gene. This aligns with current recommendations that emphasize the importance of laboratory databases and its data upload to public repositories, as this may support variant reinterpretation through allele frequency data and aggregation of phenotypic, segregation, and functional information.^57^ Nevertheless, even with supporting evidence, reclassification was not possible for 45 families with PM-VUS, which highlights the need for periodic reassessment as further data becomes available.

### Limitations

This study has several limitations. Despite comprising a large cohort with the full dataset of the national referral center for GEDs, regional founder effects, and the heterogeneous genetic background may impact solved rates and limit the external validity of our findings; furthermore, this database does not capture ethnic information, which further restricts generalizability. Although variant classification followed ACMG guidelines and incorporated FSA and clinical/bioinformatic data, a considerable number of variants remained classified as VUS. As in any real-world setting, the inability to perform additional work-up or segregation studies potentially underestimates the diagnostic yield of current molecular testing. Functional validation with in vitro splicing assays was not performed, limiting the ability to confirm pathogenicity, particularly for splicing and non-coding variants. Last, as previously discussed, both ES and targeted NGS panels have limitations, mainly the lack of coverage of non-coding regions and GC-rich exons. Although additional strategies were used when appropriate, whole-genome sequencing (WGS), reported to improve solved rate in up to 25% of negative NGS/ES cases, was not available and could have provided additional diagnosis.^58^

## Conclusions

A personalized re-evaluation approach with the thorough integration of clinical, familial, and functional research data improved the solved rate in our cohort by 10.7%, from 59.4% to 70.1%, which is on the higher end of available data.^8^ As a result, an additional 86 families (126 patients) received a definite diagnosis and, consequently, accurate data on potential therapies, prognosis, and genetic counseling. This tailored strategy incorporating advances in sequencing technology and bioinformatics tools, coupled with structured reassessment protocols under a specialized ophthalmology and medical genetics team, can substantially improve diagnostic yield in heterogeneous diseases such as GEDs. Further research on gene-disease associations, assessment of variant pathogenicity using both bioinformatic and in vitro tools, broader genome coverage assays, such as WGS, may lead to increased diagnostic yield.^59^ Furthermore, the incorporation of high-accuracy long-read technology, which is more effective in structural variant detection and haplotype phasing, while maintaining similar success rates in detecting single-nucleotide variants, and small insertions and deletions, holds promise and will be key in solving challenging cases.^60^

## Supplementary Material

Supplement 1

Supplement 2

Supplement 3
