# Supplementary material for: Enhancing Molecular Diagnostic Accuracy in Genetic Eye Disorders Through a Personalized Re-Evaluation Strategy
Source: Invest Ophthalmol Vis Sci. 2026 Jan 30;67(1):62. doi: 10.1167/iovs.67.1.62 (PMC12859735; doi:10.1167/iovs.67.1.62)
Supplement: Supplement 1 [file iovs-67-1-62_s001.docx]

**Supplemental data**

**Supplemental table 1:** Content of NGS panels according to clinical phenotype.

**Supplemental table 2:** Molecular testing results for all included partially-solved and solved cases.

**
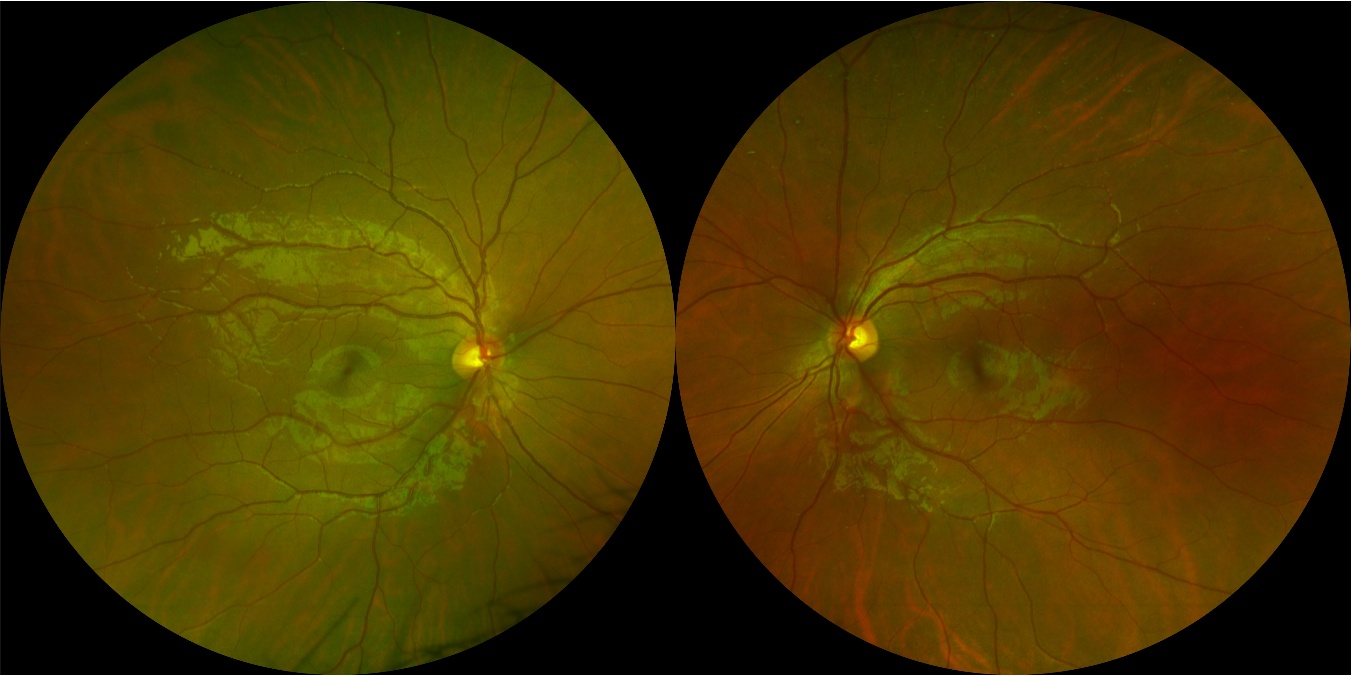
**

**Supplemental figure 1:** Widefield colour fundus photographs of P377, a heterozygous carrier of an *ABCC6* variant with a *forme-fruste* phenotype of pseudoxanthoma elasticum.
